# Editor’s introduction

**DOI:** 10.1186/s40854-022-00374-1

**Published:** 2022-07-14

**Authors:** Gang Kou

**Affiliations:** Southwestern University of Finance and Economics, Chengdu, China

The 33rd volume of Financial Innovation (FIN), Volume 8, No.3 (2022) presents 18 papers contributed by authors and co-authors from fourteen countries and areas: Australia, China, France, India, Korea, Luxemburg, Pakistan, Peru, Portugal, Saudi Arabia, Syria, Taiwan, Tunisia and USA.

- Asset pricing and trading (6 papers)

The paper “A closed-form pricing formula for European options in an illiquid asset market” addresses the problem of pricing European options. A liquidity discounting factor is first introduced and the accuracy of the formula is verified. It demonstrates the significance of incorporating liquidity risk into option pricing.

The paper “ARMA-GARCH Model with Fractional Generalized Hyperbolic Innovation” introduces a multivariate model and estimates the high-frequency returns of six U.S. stocks. The result shows that the fractional generalized hyperbolic process performs better in describing the behavior of the residual process of high-frequency returns than the non-fractional processes.

The paper “To jump or not to jump: momentum of jumps in crude oil price volatility prediction” finds a phenomenon, “momentum of jumps” (MoJ) and proposes a strategy that significantly outperforms the individual models and a series of competing strategies to enhance the predictability of crude oil price volatility.

The paper “Corporate managers, price noise and the investment factor” investigates the impact of flows between the bond and equity funds on investment factors over the period 1984 to 2015. It determines contemporaneous mispricing effects and a statistical reversal relation between these flows and both legs of the investment factor.

The paper “DeepPricing: pricing convertible bonds based on financial time-series generative adversarial networks” proposes a model to address the difficulty of valuing convertible bonds. The experiments on the Chinese convertible bond market demonstrate its effectiveness.

The paper “A theory of very short-time price change: security price drivers in times of high-frequency trading” analyzes and identifies four parameters considered to affect price changes. The study finds the volumes exchanged are not integral agents for VSTPC.

- Stock market (6 papers)

The paper “Financial Development during COVID-19 Pandemic: The Role of Coronavirus Testing and Functional Labs” analyzes the money supply reaction to the COVID-19 pandemic in 115 countries. An increasing number of recovered cases and COVID-19 testing capabilities help to increase stock trade across countries and expand financial development.

The paper “A New Analytical Approach for Identifying Market Contagion” identifies the excessive comovement of two markets as contagion. Furthermore, it applies the analytical Bayesian approach to empirically test the contagion effects of the U.S. stock market during the global financial crisis using 22 developed equity markets.

The paper “Modelling the dynamics of Stock Market in the GCC Countries: Evidence on Persistence to Shocks” implies that the long-memory dependencies in the conditional variance processes of stock market returns appear important, asymmetric, and differ in their volatility responses to unexpected shocks.

The paper “Detecting the lead–lag effect in stock markets: definition, patterns, and investment strategies” designs a strategy integrating the lead–lag strategy into classic alpha-factor strategies, based on finding from 10 years of trading data of U.S. and Chinese stock markets.

The paper “Can investors profit by utilizing technical trading strategies? Evidence from the Korean and Chinese stock markets” implies that the difference in investor behaviors between the Korean and Chinese stock markets might result in dissimilar trading strategies being employed for these two markets.

The paper “Uncertainty index and stock volatility prediction: evidence from international markets” constructs a composite UI based on the scaled principal component analysis (s-PCA) method and demonstrates its significance in- and out-of-sample predictabilities for realized variances in global stock markets.

- Corporate finance (4 papers)

The paper “Entrepreneurial, institutional and financial strategies for FinTech profitability” examines the main drivers of FinTech profitability and the time it takes for FinTech startups to break even.

The paper “Impact of CEO Attributes on Corporate Reputation, Financial Performance, and Corporate Sustainable Growth: Evidence from India” investigates the impact of CEO attributes, and provides a basis for the shareholders and companies to identify areas of consideration when appointing CEOs and determining their roles and responsibilities.

The paper “Tone of Language, Financial Disclosure, and Earnings Management: A Textual Analysis of Form 20-F” investigates the relationship between the three factors above. The results suggest that the tone used in financial disclosures and textual analysis can be informative and effective for identifying earnings management.

The paper “Near is more: learning efficiency in research and development innovation among interlocking firms” studies the performance of A-share-listed companies in China from 2007 to 2017. The results support that the information obtained by interlocking directorates through external social relations guides firms’ decision-making, and closer distances reveal more obvious effects.

- Financial intermediation (2 papers)

The paper “Does microfinance foster the development of its clients? A bibliometric analysis and systematic literature review” analyzes the trends in microfinance outcomes from the perspective of their recipients, specifically more vulnerable people, while also focusing on the demand side.

The paper “Consumer lending efficiency: commercial banks versus a fintech lender” compares the performance efficiency of LendingClub’s unsecured personal loans with similar loans originated by banks. The results show that LendingClub’s increased use of alternative data and AI/ML may have improved its credit risk assessment capacity above and beyond its peers using traditional approaches.

